# Population Analysis of Masseter Muscle Tension Using Shear Wave Ultrasonography across Different Disease States

**DOI:** 10.3390/jcm13175259

**Published:** 2024-09-05

**Authors:** Rafal Obuchowicz, Barbara Obuchowicz, Karolina Nurzynska, Andrzej Urbanik, Malgorzata Pihut

**Affiliations:** 1 Department of Diagnostic Imaging, Jagiellonian University Medical College, 30-663 Krakow, Poland; 2 Department of Conservative Dentistry with Endodontics, Jagiellonian University Collegium Medicum, Montelupich 4, 31-155 Cracow, Poland; 3 Institute of Informatics, Faculty of Automata Control, Electronics, and Computer Science, Silesian University of Technology, Akademicka 16, 44-100 Gliwice, Poland; 4 Prosthodontic and Orthodontic Department, Dental Institute, Jagiellonian University Medical College, 31-155 Krakow, Poland

**Keywords:** masseter muscle, shear wave, temporomandibular disorders, elastography

## Abstract

**Objective:** This study aimed to evaluate the distribution and trends of masseter muscle tension in patients with temporomandibular joint (TMJ) pain, examining gender-specific differences and the impact of various TMJ disorders. **Methods:** From January 2020 to June 2024, a total of 734 patients presenting with facial pain radiating to the head and neck, localized around and extending from the TMJ, were referred for ultrasonographic examination. After applying exclusion criteria, 535 patients (72.9%) were included in the study. The patient cohort consisted of 343 females (64.1%) and 192 males (35.9%), with muscle tension measured using the Aixplorer ultrasound system equipped with a shear wave device. Data were collected and analyzed across different age groups and TMJ conditions, including “no changes”, “exudate”, “arthrosis”, and “disc displacement”. **Results:** The study found that males exhibited higher muscle tension across all conditions, particularly in the “no changes” (40.4 kPa vs. 32.1 kPa, 25.9% higher) and “exudate” (38.5 kPa vs. 29.7 kPa, 29.6% higher) categories, indicating increased muscle strain and inflammation during middle age. In females, a trend of decreasing muscle tension with age was observed, with a significant reduction from 36.2 kPa in the 20–30 age group to 24.3 kPa in the 60–70 age group (32.9% reduction), suggesting a reduction in muscle mass or strength due to aging. Both genders showed high muscle tension in the presence of exudate, with females peaking in the 40–50 age group at 37.1 kPa and males peaking earlier in the 20–30 age group at 41.2 kPa (10.9% higher in males), highlighting potential gender differences in inflammatory response. In the arthrosis group, males displayed a consistent increase in muscle tension with age, peaking at 37.5 kPa in the 50–60 age group (50.7% increase from the 20–30 age group), while females showed high tension, particularly in the 40–50 age group at 31.0 kPa (82.4% higher compared to the 20–30 age group), indicating the need for targeted joint health interventions in middle-aged women. **Conclusions:** This study reveals significant gender-specific differences in masseter muscle tension among patients with TMJ pain. Males were found to be more affected by muscle strain and inflammation during middle age, whereas females showed a significant decrease in muscle tension with age. The presence of exudate significantly impacted muscle tension across all age groups for both genders. These findings underscore the importance of tailored clinical interventions and preventive strategies to manage TMJ disorders effectively.

## 1. Introduction

Optimal functional and anatomical congruence among the teeth, supporting tissues, and temporomandibular joints is achieved under the efficient control of muscles and nerves. Coordination of these structures is essential to ensure the health, functional efficiency, and stability of the entire stomatognathic system [1]. Approximately 60–70% of the general population exhibit signs of temporomandibular disorders (TMD), yet only about 25% of those individuals are aware of or report symptoms [2]. The prevalence of severe temporomandibular disorders, which are accompanied by headache and facial pain, is observed in 5–12% of the adult population [3].

Contributing factors to TMD include occlusal abnormalities, functional shift, parafunctional habits such as bruxism, orthopedic instability, and microtrauma. Joint laxity also plays a role, similar to occlusal overloading and increased joint friction. Psychosocial factors, including stress, anxiety, and depression, may also lead to TMD [4,5,6]. Patients with condylar dysmorphism, especially those with steep articular eminences, are more likely to exhibit significant condyle-disk movement during function. This excessive movement may elevate the risk of ligament elongation, which can lead to disk derangement disorders [7]. Regarding the distribution of occlusal contacts, the symmetry of contact intensity, rather than the symmetry of contact number in the posterior occlusion, appears to be more significant in relation to temporomandibular function [8].

The most frequent clinical features of TMD are chronic myofascial pain, masticatory muscle pain, and a limited range of mouth opening. Accompanying symptoms, such as earache, headache, toothache, and neuralgia in the head region, may also be present [9,10,11]. In the present study, we focus on the masseter muscle as a critical component of the stomatognathic system. Inappropriate functioning and stiffness of the masseter muscle can result in various pathologies, including myalgia, myofascial pain, and disordered function [12]. Bite forces significantly impact the masticatory muscles. Specifically, the masseter muscles contribute significantly to stabilizing the grinding path during mastication [13,14]. Clinicians emphasize the importance of addressing increased stiffness and tonus of the masseter muscle as part of the conservative treatment of TMD [15].

Shear wave elastography (SWE) uses focused ultrasound pulses to generate a localized acoustic radiation force within the tissue. This force induces a minute, temporary displacement in the tissue, creating shear waves that propagate perpendicularly to the direction of the ultrasound pulse. The shear waves generated within the tissue are then detected using longitudinal ultrasonography waves, which travel faster than the shear waves and are used to capture the displacement caused by the shear waves. The Aixplorer device uses algorithms to track the speed and propagation of shear waves in real time. By measuring the speed at which these waves travel through the tissue, the device can compute the tissue’s stiffness. The result is a quantitative, real-time map of the elastic modulus (a measure of tissue stiffness) across the scanned tissue area. The stiffness values are expressed in kilopascals (kPa), providing an objective measure of tissue mechanical properties [16,17,18]. SWE measurements are influenced by different variables that impact the consistency and accuracy of SWE measurements., such as the unit of measurement, the depth of the muscle tissue, and the load applied by the ultrasound probe [19]. The role of SWI in a measurement of muscle stiffness is well established, with a potential to detect different muscle-related anomalies [20,21,22]. Maps of muscle tension distribution were created also with special interest in muscle tension involved with pain in the head region [23]. 

While the literature discusses the challenges of measuring muscle tension in the orofacial region and the impact of increased muscle tension on TMJ function, a significant gap remains. Specifically, there is a lack of population-level studies that explore the distribution of elevated masseter muscle tension in relation to various common TMJ-related morbidities [24,25,26,27]. This research is critically important because understanding these trends on a broader scale can lead to more effective, tailored interventions for individuals suffering from TMD. By addressing this gap, the present study contributes valuable insights into the epidemiology of masseter muscle tension and its implications for TMJ health, thereby informing better clinical practices and improving patient outcomes.

The primary aim of this study was to evaluate population-level changes in masseter muscle stiffness among individuals experiencing temporomandibular joint (TMJ) pain and TMJ-related pain resulting from joint dysfunction. This objective is driven by the need to better understand the role of masseter muscle stiffness in the etiology and progression of TMJ disorders, which are common yet often underdiagnosed conditions affecting the masticatory system.

## 2. Method

From January 2017 to June 2022, 734 patients in the process of TMJ pain evaluation were referred for ultrasonographic temporomandibular joints evaluation. The ultrasonographic diagnostic processes of patients who fulfilled criteria of inclusion for the study referred from different clinical departments of the Clinical Hospital were held in the Department of Radiology and Ultragen medical clinic. To ensure repeatability of the results, a radiologist with at least six years’ experience in the ultrasonographic examination process with use of shear wave technology was personally involved in the measurements and personally responsible for the process. Institutional Ethics Committee of the Jagiellonian University no. 1072.6120.138.17. 

Patients presenting with facial pain radiating to the head and neck, localized around and extending from the temporomandibular joint (TMJ), were referred for ultrasonographic examination. This group consisted of patients referred for an initial ultrasound (US) examination as part of the diagnostic process.

### 2.1. Inclusion and Exclusion Criteria

Inclusion Criteria were as follows:

1. Age range: Patients aged between 20 and 70 years, covering a broad demographic to evaluate age-related trends in masseter muscle stiffness.
2. Presence of TMJ pain: Individuals experiencing temporomandibular joint (TMJ) pain or TMJ-related pain radiating to the head and neck, localized around and extending from the TMJ.
3. Diagnosis of TMJ dysfunction: Patients with clinical signs of TMJ dysfunction, such as disc displacement, arthrosis, or exudate in the joint, as confirmed by ultrasonographic examination.
4. Masseter muscle evaluation: Participants who are suitable for shear wave elastography (SWE) to assess masseter muscle stiffness, ensuring that their condition allows for accurate ultrasonographic measurement.
5. Symptomatic individuals: Patients reporting persistent TMJ pain, even in the absence of visible ultrasonographic changes, to explore potential psychosomatic contributions to muscle stiffness.

The exclusion criteria were as follows:

1. Patients with inflammatory diseases or chronic infections (e.g., Borrelia infections)—as factors which can alter muscle tension values.
2. Patients with recent trauma—trauma can change muscle tension with mechanical influence on muscle structure.
3. Patients presenting with diagnosed or visible neurological diseases—changes in efferent nervous signals influences muscle tension.
4. Patients who had undergone surgery in the facial/neck region—iatrogenic muscle trauma can influence muscle structure.
5. Patients using analgesics or muscle relaxants—drugs which directly influence muscle function changes muscle tension values.
6. Patients who had recently undergone physiotherapy—physiotherapy is usually directed towards muscle tension decrease.

After applying the exclusion criteria, 535 patients were included in the study. Before the examination, patients completed a self-assessment questionnaire and provided informed consent. Patients were informed about the procedure, emphasizing its non-invasive nature and examination strategy. 

This protocol was approved by the institutional review board and approved by the Institutional Ethics Committee of the Jagiellonian University no. 1072.6120.138.17 dated 28 September 2017. The US procedure and patient management adhered to Good Medical Practice and the Helsinki Declaration.

### 2.2. Examination Procedure

Patients were positioned supine and relaxed in habitual occlusion; any splints were removed. Gel was applied to the skin, and the patient was examined using the Aixplorer ultrasound system equipped with a shear wave device (Aixplorer, Aixen Provence, France), utilizing a 5–12 MHz linear-array probe (SL10-2, Aixplorer [SuperSonic Imagine]).

The temporomandibular joint was examined using morphological sections parallel to the joint line. This approach allowed for the visualization of exudate, irregularities of the bony surface, overall joint congruence (i.e., the relative positions of joint surfaces), and the position of the disk during habitual occlusion and dynamic examination during jaw movement (opening and closing the mouth on command).

### 2.3. Muscle Tension Measurement

**Figure 1:**
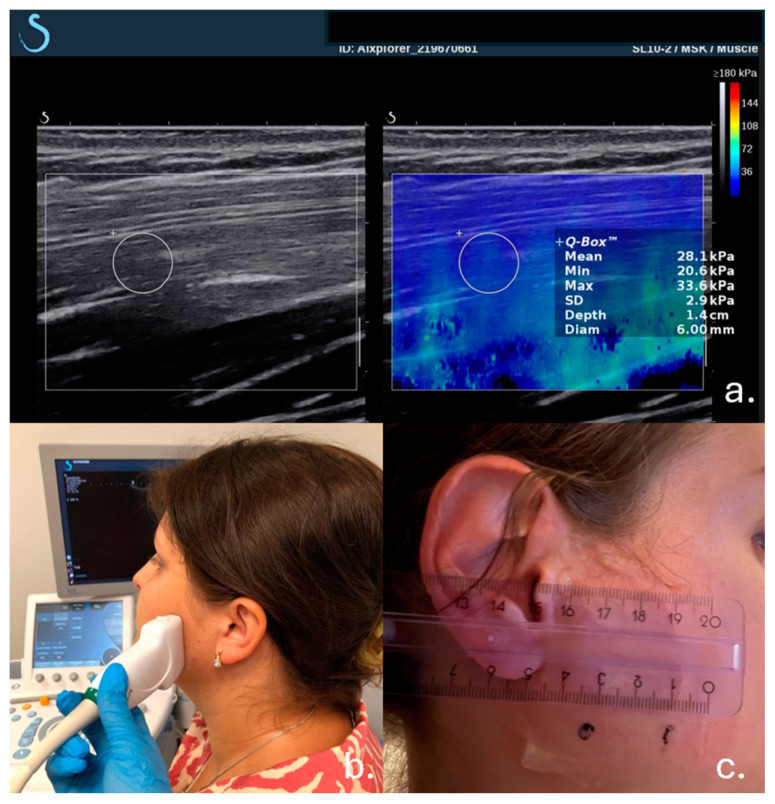
An example of ROI placement of the masseter muscle with readings of muscle tension (**a**). Position of the probe during examination of the patient (**b**). Distance between black dots where the probe was placed (**c**).

Muscle tension was measured with the mouth closed in a comfortable supine position. The transducer was placed against the patient’s face over the zygomatic arch in the anterosuperior compartment of the joint, sagittal to the frontal plane, 22 mm above the visualized inferior border of the mandible. The entire muscle was visualized between the outer fascia and the surface of the mandibular ramus, with minimal possible tension applied during measurement (Figure 1). SWE measurements were provided with adjustable ROI of 6 mm, an ROI called Q-box. Inaccurate measurements were indicated by several factors, including suboptimal ultrasonographic imaging (bad quality B-image), out-of-range measurements, and improper marking of the measurement region of interest (ROI) by color. These phenomena were considered critical indicators of potential measurement imprecision.

Three measurements were taken from both sides (left and right), and the mean value was recorded. Potentially inaccurate measurements were not included to the study. Recorded data were analyzed statistically (Statistica program).

### 2.4. Data Collection and Analysis

Morphological and functional data were collected from both the left and right joints as per standard examination protocol. The prevalence of unilateral and bilateral occurrence of the disease across age groups was analyzed (details presented in the Supplementary Materials). For clarity, muscle tension data were analyzed for patients with unilateral predominance of the most prevalent conditions diagnosed via ultrasonography. The “no changes” group included patients with no visible ultrasonographic changes; the “exudate” group included patients with dominant exudate in the joint; the “arthrosis” group included patients with dominant joint degeneration; and the “disc displacement” group included patients with disc displacement.

**Figure 2:**
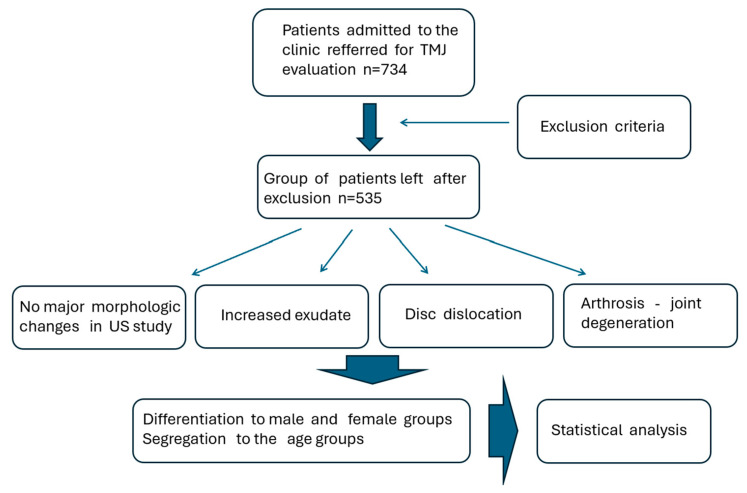
Scheme presenting the plan of the study and data management (area of statistical analysis).

Statistical analyses were meticulously carried out using the STATISTICA software package (Tibco) version 13.3, a comprehensive tool widely recognized for its robustness in data analysis. The analyses began with the computation of descriptive statistics, which provided a detailed summary of the central tendencies, dispersion, and overall distribution of the data, as illustrated in the subsequent scheme (Figure 2). To ensure the appropriateness of parametric methods, the Shapiro–Wilk test was applied to assess the normality of the data distribution across the groups.

For evaluating differences between groups with different pathologies and no pathology presented, the non-parametric Mann–Whitney test was chosen, owing to its effectiveness in comparing two independent groups when the assumption of normality is violated. This test allowed for a precise comparison of the medians offering insights into any statistically significant differences.

Moreover, to explore the relationships between categorical variables, particularly concerning temporomandibular disorders (TMD), chi-squared tests were utilized. These tests were essential for analyzing the association between the groups, enabling the examination of frequency distributions and independence between variables. This multifaceted statistical approach ensured a thorough and reliable analysis of the data, providing a robust basis for interpreting the findings.

### 2.5. Studied Patients Group

Table 1 provides data that reflect a higher representation of younger participants, particularly females in the 20–30 age group and males in the 30–40 age group. The lower number of older participants, particularly in the 60–70 age range, may impact the generalizability of findings across all age groups. The difference in gender distribution across age groups could indicate highly varied levels of the prevalence of TMJ-related conditions among different genders at different ages.

**Figure 3:**
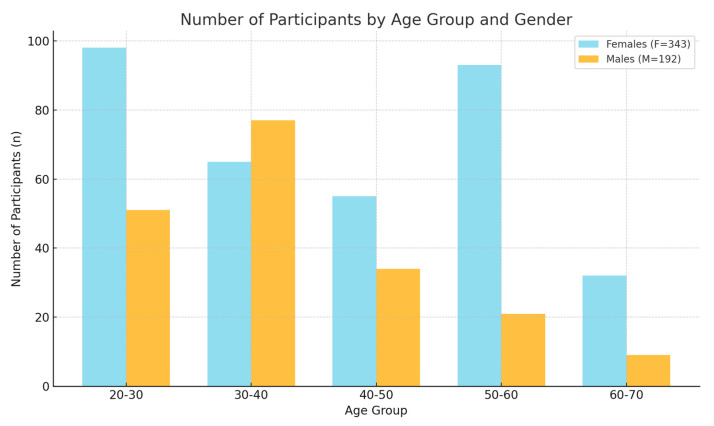
The distribution of patient numbers in different age groups (five from 20 yo. to 70 yo.) of age groups. Male and female are compared side by side (light blue and red, respectively).

This graph (Figure 3) presents age and gender differences, showing distinct differences in the distribution of participants by age and gender. Notably, there was a peak in female participants in the 50–60 age group, whereas the male participants peaked in the 30–40 age group. Age-related trends: The number of participants declined with age for both genders, but the pattern of decline was different for males and females, indicating potential age-related factors affecting participation or the prevalence of the condition being studied. 

Results:

Muscle tension values in females with arthrosis rose from 17 kPa in the 20–30 age group, peaked at 31 kPa in the 40–50 age group, and then slightly decreased to 24 kPa in the 60–70 age group. In comparison, males show higher initial tension (25 kPa) and peak tension (37 kPa) values. This indicates that while arthrosis impacts both genders significantly, it tends to cause more pronounced increases in muscle tension in males, particularly during middle age.

For disc displacement, females show relatively stable muscle tension across age groups, ranging from 17 kPa to 30 kPa, with a peak in the 40–50 age group. Males, on the other hand, display higher and more variable tension values (ranging from 20 kPa to 35 kPa). This stability in females may reflect different biomechanical responses to disc displacement compared to males, where disc displacement might lead to more significant fluctuations in muscle tension (Table 2 and Table 3).

Statistical significance in the presented data: 

Gender differences in muscle tension: Exudate condition (20–30 Age Group): Males show significantly higher muscle tension compared to females (*p*-value: <0.01), which may indicate that younger males experience greater muscle tension due to inflammation.

In the female cohort:

Exudate condition (40–50 age group): Muscle tension significantly increased in the 40–50 age group compared to the 30–40 age group (*p*-value: <0.05).

Arthrosis condition (40–50 age group): Significant increase in muscle tension from the 20–30 to the 40–50 age group was observed (*p*-value: <0.05).

Disc displacement group: Muscle tension increases were not statistically significant across age groups (*p*-value: >0.05).

In the male cohort:

No changes condition (40–50 age group): Significant increase in muscle tension from the 20–30 to the 40–50 age group (*p*-value: <0.05).

Exudate condition (20–30 age group): A significant difference in muscle tension between the 20–30 and 50–60 age groups was observed (*p*-value: <0.01).

Arthrosis condition (50–60 age group): A significant increase in muscle tension from the 40–50 to the 50–60 age groups was observed (*p*-value: <0.05).

Disc displacement (40–50 age group): A significant increase in muscle tension between the 30–40 and 40–50 age groups was present (*p*-value: <0.05).

Decline in Muscle Tension with Aging: Exudate Condition (males, 60–70 age group): Presented a significant decline in muscle tension from the 20–30 to the 60–70 age groups (*p*-value: <0.01). The decline in muscle tension with aging was not statistically significant in the female group (*p*-value: >0.05).

There was a gradual increase in muscle tension in the female group, peaking during the middle age range (40–50 years). This increase was followed by a noticeable decrease in muscle tension in the older age groups (50–60 and 60–70 years). Peak Tension: The highest muscle tension was observed in the 40–50 age group, after which there was a clear decline as age increased. This suggests that muscle tension increases with age up until middle age, possibly due to factors like increased muscle use or stress, and then decreases, potentially due to muscle weakening or other age-related changes.

In the male group, muscle tension was consistently higher compared to the female group across all age groups. The tension increased significantly during the younger and middle ages, peaking in the 40–50 age group.

Comparison with females: Males exhibited higher muscle tension than females, particularly in the younger (20–30 years) and middle age (30–50 years) groups. This heightened tension could be attributed to factors such as greater muscle mass, differences in physical activity levels, or stress-related impacts on males during these life stages.

Peak tension: Similar to females, the peak muscle tension for males occurred in the 40–50 age group, which may be a critical period for stress and muscle strain, after which a decline was observed, although the decline was less pronounced compared to the female group.

**Figure 4:**
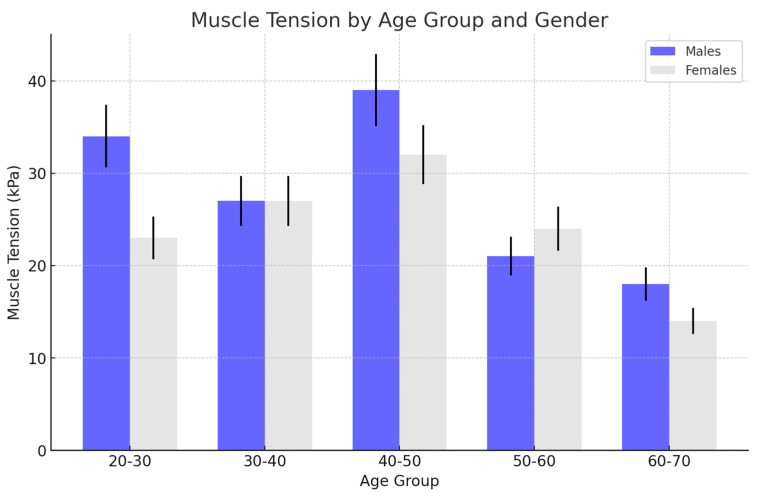
The changes in muscle tension across different age groups for females and males (light grey and blue), which presented no visible changes in the ultrasonographic picture in the female group. In the female group, there was a gradual increase in muscle tension until middle age (40–50), observed then with a decrease in older age. In the male group, there was higher muscle tension in comparison to women, which was observed in younger and middle-aged groups, peaking at 40–50 years.

The following graph (Figure 4) presents some implications. Gender differences: The figure highlights a clear gender difference in muscle tension, with males showing consistently higher tension across all age groups. This could suggest that male muscles are under greater strain or that they respond differently to the same stimuli compared to females.

Both genders show a peak in muscle tension around the 40–50 age range, which might be associated with lifestyle factors, increased responsibilities, or the cumulative effects of stress over time. The subsequent decline in muscle tension in older age groups for both males and females could be due to natural aging processes, such as the loss of muscle mass or reduced physical activity.

**Figure 5:**
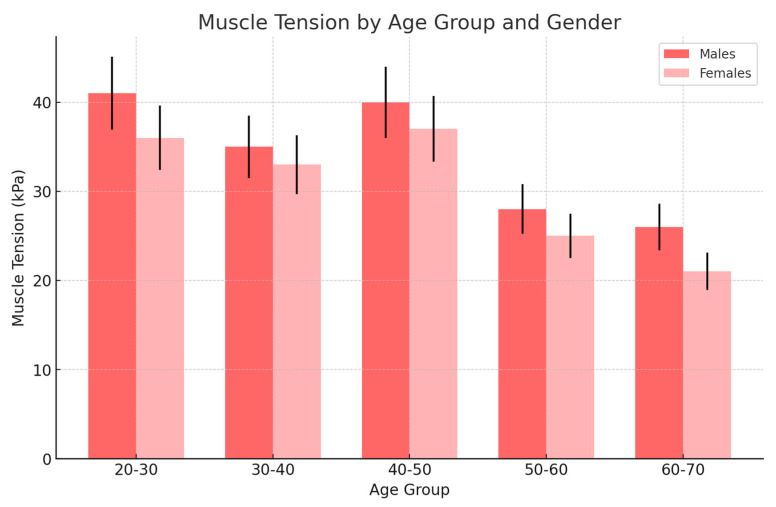
The changes of muscle tension across different age groups for the female and male groups (light red and red, respectively), which presented exudate (inflammation) in the ultrasonographic picture. In the case of this condition, in the female group, high muscle tension due to inflammation peaked in younger (20–30) to middle age (40–50) and then decreased. However, in men with inflammation, consistently high muscle tension was observed with a peak in younger age (20–30) and marked decrease in older age (60–70).

In females, muscle tension was found to be elevated due to inflammation, with values peaking between the younger age group (20–30 years) and middle age (40–50 years). This trend suggests that inflammation-induced muscle tension is most significant during early to middle adulthood (Figure 5). Peak tension: The highest muscle tension was observed in the 40–50 age group, with a subsequent decline as age increased. This decline in muscle tension in older age groups may reflect a reduction in the inflammatory response or a decrease in muscle mass or activity as women age.

In males, consistently high muscle tension was observed across the younger age groups, particularly peaking in the 20–30 age group. Unlike females, where muscle tension decreases gradually with age, males were shown to exhibit a more pronounced and consistent decline in muscle tension as they age, especially noted in the 60–70 age group. Peak tension: The peak in muscle tension for males occurred earlier, in the 20–30 age group, indicating that younger males with exudate may experience the highest levels of muscle strain due to inflammation. This suggests that inflammation may have a more intense effect on muscle tension in younger males, which could be linked to higher levels of physical activity or other stress-related factors during this life stage.

Gender differences: The figure highlights significant gender differences in how inflammation (exudate) affects muscle tension across age groups. While both genders show increased muscle tension due to inflammation, the timing and magnitude of these peaks differed. Females tended to peak later in middle age, while males peaked earlier and then experienced a marked decrease in older age. The data suggest that inflammation has a strong impact on muscle tension in both younger and middle-aged adults, but this effect diminishes in older age, particularly in males. The decline in muscle tension in older males could be due to reduced inflammatory response or a decrease in muscle function with aging.

**Figure 6:**
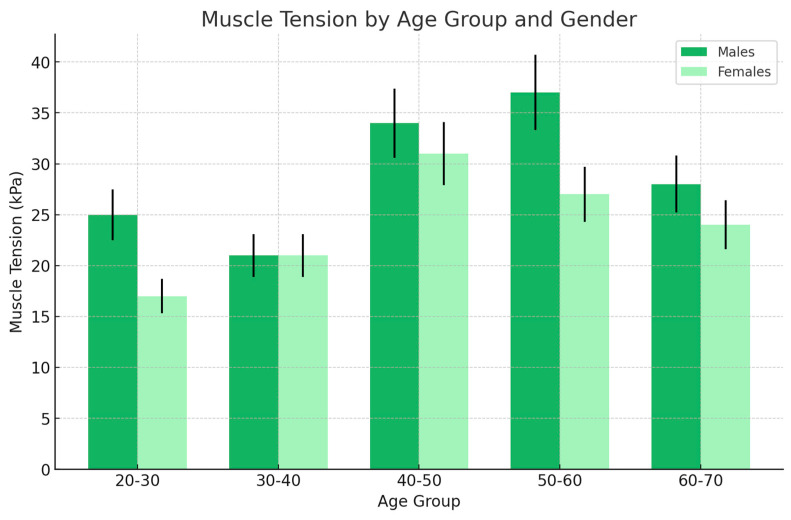
The changes of muscle tension across different age groups for the female and male groups (light green and green, respectively), which presented arthrosis (degeneration) in the ultrasonographic picture. In the female group, muscle tension increased with age, peaking at 40–50, which may correlate with the progression of joint degeneration. In the male population, higher muscle tension in younger and middle-aged groups in comparison to women was observed, peaking at 50–60, probably with correlation with severe joint debridement.

In females, muscle tension increased with age, reaching its peak in the 40–50 age group. This trend suggests a correlation between advancing age and the progression of joint degeneration, where muscle tension might increase as arthrosis worsens (Figure 6). The highest muscle tension in females was observed in the 40–50 age group. After this peak, there was a slight decline in the 50–60 age group and a more noticeable decline in the 60–70 age group, possibly due to reduced muscle function or the natural weakening of muscles as aging progresses. In males, muscle tension was higher in the younger (20–30) and middle-aged (30–60) groups compared to females, with a peak in the 50–60 age group. The increased tension during these life stages may be associated with more severe joint debridement or higher levels of physical activity contributing to joint stress. The peak in muscle tension for males occurred later than in females, at the 50–60 age period. This may indicate that in males, the effects of arthrosis become more pronounced at a slightly older age, leading to increased muscle tension as the joint degeneration progresses.

The figure highlights that males generally exhibited higher muscle tension across most age groups compared to females when arthrosis was present. The later peak in males suggests that joint degeneration might have a more gradual but sustained impact on muscle tension in this gender. Both genders show an increase in muscle tension correlating with the progression of arthrosis. However, the age at which this peak occurred differed between males and females, reflecting potentially different patterns of disease progression or physical activity levels that exacerbate joint degeneration.

**Figure 7:**
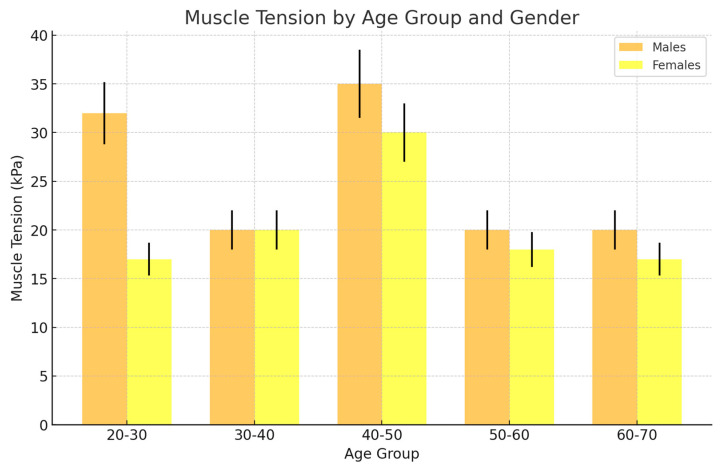
The changes of muscle tension across different age groups for the female and male groups (yellow and orange, respectively), which presented disk displacement in the ultrasonographic picture. In the male cohort, increased tension was observed in comparison to the female group, with a peak in younger and middle age in females, with joint instability presenting less impact on muscle tension increasing but being relatively stable thought life.

In the female group, muscle tension remained relatively stable across most age groups, with no significant peaks or declines. This suggests that disc displacement has a consistent but moderate impact on muscle tension throughout life (Figure 7). The highest muscle tension for females occurred in the 40–50 age group. However, the overall variation in muscle tension across different ages was minimal, indicating that joint instability due to disc displacement in females has a limited impact on increasing muscle tension.

In contrast to females, males exhibited higher muscle tension across the younger and middle-aged groups. The tension peaked in the 20–30 and 40–50 age groups, indicating that younger and middle-aged males experience the most significant impact of disc displacement on muscle tension.

The peak muscle tension for males was observed in the 40–50 age group, similar to females, but the overall muscle tension values were higher in males than in females, especially in the younger age groups.

The figure highlights that males generally have higher muscle tension associated with disc displacement compared to females, especially in the younger and middle-aged groups. This could be due to factors like higher physical activity levels, greater muscle mass, or different biomechanical responses to joint instability in males. While both genders show a peak in muscle tension around the 40–50 age group, males experienced more pronounced increases in tension earlier in life (20–30 years). This suggests that disc displacement might lead to more significant functional impacts on the masticatory muscles in males during these critical life stages.

**Figure 8:**
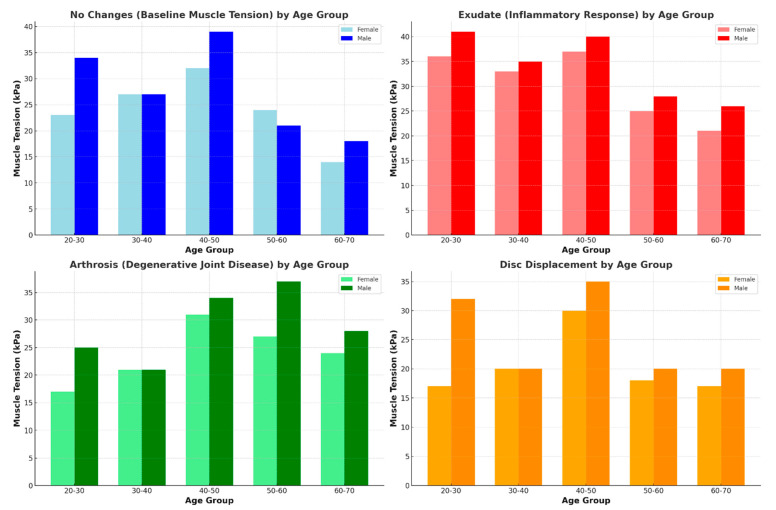
A summary of the influence of the different conditions across females and males of different ages.

On the basis of the following data, the following summarizing comments can be made in the female population with no visible US changes (Figure 8) muscle tension decreased with age, from 23 kPa in the 20–30 age group to 14 kPa in the 60–70 age group. In the male population, muscle tension remained relatively high across age groups, peaking at 39 kPa in the 40–50 age group and reducing to 18 kPa in the 60–70 age group. 

In the group with dominant exudate (inflammatory response) in the female population, muscle tension was markedly increased in comparison to other groups and peaked at 37 kPa in the 40–50 age group; in the 60–70 age group, muscle tension decreased to 21 kPa. In the male population, muscle tension was importantly increased, peaking at 41 kPa in the 20–30 age group. An important decrease to 26 kPa in the 60–70 age group was observed.

In the group representing arthrosis (degenerative joint disease) in the female population, muscle tension increased with age, peaking at 31 kPa in the 40–50 age group, and then slightly decreased in the older age group. In the male population, it was importantly increased to 37 kPa in the 50–60 age group and remained high (28 kPa) in the 60–70 age group.

In the group presenting disc displacement as a dominant morbidity in the female population, muscle tension was increased but relatively consistent across age groups, with slight decreases in older age groups. In the male population, muscle tension was highest in the 20–30 age group, reaching 32 kPa, and remained high in the 40–50 age group at 35 kPa, with a decrease in the older age group.

Understanding these trends is crucial for developing gender- and age-specific treatment plans for managing TMJ disorders associated with inflammation arthrosis and disk dislocation. For example, younger males may require more aggressive anti-inflammatory interventions, while middle-aged females might benefit from therapies that address both inflammation and its long-term effects on muscle tension. In the patients with arthrosis, interventions might need to be tailored according to age and gender, with males possibly requiring more aggressive treatment during middle age to manage muscle tension and joint health, while females might benefit from earlier interventions. The relatively stable muscle tension in females with disk displacement across age groups suggests that they might be less affected by the functional consequences of disc displacement compared to males. For males, particularly those in younger and middle age, more aggressive management strategies might be necessary to mitigate the impact of disc displacement on muscle tension and overall TMJ function.

## 3. Discussion

In our study, clear trends were identified in the distribution of masseter muscle tension in the population, with separate analyses for female and male populations. 

The most extreme muscle tension values indicating conditions of the highest clinical importance were found in this study. In the female group with excessive exudate, muscle tension peaked in the 40–50 age group at 37 kPa, which may correlate with increased inflammatory responses during middle age. With arthrosis, peak tension also occurred in the 40–50 age group at 31 kPa, indicating that joint degeneration has a significant impact on muscle tension during middle age. In the group where disc displacement was observed, tension remained relatively stable but peaked in the 40–50 age group at 30 kPa, showing moderate increases due to joint instability.

In the male group with no visible findings, muscle tension peaked in the 40–50 age group at 39 kPa, suggesting a general increase in muscle strain during middle age, even without visible joint changes. In the group with excessive exudate, peak tension occurred earlier, in the 20–30 age group at 41 kPa, indicating that younger males experience significant muscle tension due to inflammation. In the group with arthrosis, muscle tension peaked in the 50–60 age group at 37 kPa, correlating with the progression of joint degeneration in older age. In the group with disk displacement, the peak muscle tension occurred in the 40–50 age group at 35 kPa, indicating that joint instability significantly affects muscle tension during middle age.

The prevalence of the female population in our study aligns with observations from other researchers [28,29]. The literature suggests that the number of females seeking attention for orofacial pain outnumbers males by as much as 8:1 [30]. However, our findings indicate a female-to-male prevalence ratio of approximately 2:1 (343 females to 192 males). It is notable that in the general population, the prevalence of TMJ disorders is reported to be equal between females and males, although females report the condition more frequently [31].

Males exhibited higher muscle tension across all conditions, particularly in the groups with no evident changes and those with exudate, suggesting increased muscle strain and inflammation during middle age. Although direct comparisons to our findings are challenging due to the lack of literature on the distribution of muscle tension in the general population, our observations in the investigated patient group align with the work of Olchowy [32], who reported a significant difference in baseline muscle tension between women and men, with a 9% difference. Dietisch also emphasized the reduction in muscle tone in women compared to men [33].

Olchowy proposed baseline tension values in the normal population, with approximately 10 kPa for both genders, serving as reference values. In our study, the presented values were higher, even in the group with no visible ultrasonographic changes. However, our study did not include healthy subjects but rather patients referred with persistent TMJ pain. As noted by Voscopulous, underlying morbidity must be suspected as a one of factors in patients with persistent TMJ pain [34].

In the group of ultrasonographically “normal” joints, particularly in younger patients, psychosomatic conditions can be suspected as frequent important factors for increased muscle tension, especially in the orofacial region [35,36,37,38,39,40,41]. This may explain the numerous patients presenting with symptoms of head pain without a clear morphological cause.

Our study found a close correlation between the increased amount of fluid and increased muscle stiffness. Exudate in the joint, known to be an effect of ongoing inflammation, might be a provoking factor for increased masseter stiffness, related at least in part to neural reflex stimulation and the action of proinflammatory factors such as kinins [42]. This mechanism may explain the markedly increased muscle tension observed in both males and females with joint effusion. However, Afuriach [43] found no influence of rheumatoid arthritis on muscle tissue tone assessed with SWE, which might be due to differences in the investigation area, mechanics of the examined larger joints, and the specific nature of rheumatoid arthritis. In our study, patients with inflammatory diseases were excluded.

We also found a correlation between anterior disc displacement and increased muscle tension, which was likely due to jaw malposition and distortion of the musculo-ligamentous balance. This finding aligns with studies suggesting that muscle tension is partially caused by an imbalance in the stomatognathic system [44,45].

In our study, arthrosis was identified as an important factor influencing changes in muscle tension. This might result from both mechanical irritation due to osteophytosis and the influence of proinflammatory factors produced by irritated tissue. The kinin system is known to deteriorate muscle function, and in acute states, it can promote a muscle signaling cascade of inflammation, resulting in increased tension [46,47]. Our study primarily considered patients with pain, consistent with literature indicating increased muscle tension in symptomatic patients compared to healthy individuals [26], particularly around joints affected by osteoarthrosis [48].

In the examined population, we observed a gradual increase in muscle tension with age, peaking in the fourth and fifth decades, followed by a decrease in older age. This trend may reflect muscle deterioration associated with osteoarthrosis progression and aging [49,50]. All studied factors affecting TMJ health, including exudate, arthrosis, and disk displacement, impacted muscle tension, particularly in middle-aged individuals of both genders. However, a decrease in muscle tension was observed in the older age groups (sixth and seventh decades), consistent with observations in muscle biology, where aging leads to decreased muscle volume and reduced muscle contraction [51,52,53,54]. Additionally, neuromuscular dysfunctions and prolonged TMJ dysfunction, impairing masticatory function, may contribute to the gradual deterioration of masticatory muscle function and muscle tone, particularly in the masseter [55,56].

Future studies should extend the examination beyond the masseter muscle to include other masticatory muscles, such as the temporalis, medial pterygoid, and lateral pterygoid. This will provide a more comprehensive understanding of muscle tension and its role in temporomandibular joint (TMJ) disorders. To enhance the generalizability of findings, future research should involve diverse populations, including various ethnic groups and geographical regions. This would help determine whether the trends observed in this study are consistent across different demographic groups. Future research should compare shear wave elastography (SWE) with other diagnostic methods, such as magnetic resonance imaging (MRI) and electromyography (EMG), to validate findings and explore discrepancies between different technologies.

This study has potential further practical applications. The findings from this study can inform the development of personalized treatment plans that consider individual variations in muscle tension based on gender, age, and specific TMJ conditions. Clinicians could tailor interventions to target the most affected muscle groups, improving treatment outcomes. The use of SWE in this study demonstrates its potential as a non-invasive diagnostic tool for assessing muscle tension in TMJ disorders. Future applications could involve integrating SWE into routine clinical practice, providing real-time, quantitative data to support diagnosis and monitor treatment progress. Understanding the patterns of muscle tension in different populations could lead to the development of preventive healthcare strategies aimed at reducing the risk of TMJ disorders. These strategies could include early interventions for individuals at higher risk, such as those with high baseline muscle tension or significant psychosomatic stress. The study’s findings highlight the importance of addressing muscle tension in the management of TMJ disorders. This could lead to the development of new therapeutic interventions, such as targeted physical therapy, relaxation techniques, and stress management programs designed to reduce muscle tension and alleviate TMJ-related symptoms. Given the complex interplay between physical and psychological factors in TMJ disorders, future applications could involve integrating multidisciplinary approaches that combine dental care, physical therapy, and mental health support to address the full spectrum of factors contributing to TMJ dysfunction. 

Although we tried to develop a comprehensive study, there were some recognizable weaknesses in our approach. In our study, only the masseter muscle was examined. However, it is well known that tension in other muscle areas may play a significant role in provoking TMJ and TMJ-referred pain [57,58], something that might be addressed in future works. Although the patient group was relatively large, detailed analysis resulted in smaller subgroups. These changes should be studied in larger groups of patients. Our population was limited to a Caucasian cohort from the southern Poland region, and the study should be extended to different populations for broader applicability. We did not compare our results with other methods; as shown by Haueise [26], there is a marked discrepancy between reported values depending on the system used.

Examiner blinding was not utilized in this study. Muscle tension and joint condition assessments were conducted simultaneously, which may have introduced some level of bias into the results. The absence of blinding could potentially influence the examiner’s assessment, especially in conditions where subjective interpretation is required. In cases where the examiner is aware of the participants’ conditions or other variables, there may be an unintentional influence on the measurement process, particularly in assessing conditions like exudate or arthrosis. While the use of objective tools like ultrasonography mitigates some of this risk, future studies would benefit from incorporating examiner blinding to reduce bias and enhance the validity and reliability of the study outcomes.

A clear strength of this study is the presentation of muscle tension trends in a representative population across different clinical states and both genders. As far as we know, this is the first study to highlight this important aspect of TMJ dysfunction, providing valuable insights into the provoking factors of TMJ.

## 4. Conclusions

The data highlight significant gender- and age-related differences in muscle tension across various clinical conditions. Understanding these trends allows healthcare providers to develop targeted interventions and preventive strategies, ultimately improving patient outcomes and quality of life for both female and male populations. Clinical considerations regarding therapeutic strategies are not described in this work but can be found in the broader literature on the subject.

## Figures and Tables

**Table 1:** The number of participants in the study. F—(female group) and M—(male group), with the specification of age groups.

	Age Group	M = 192 Participants (N)	M = 192 Participants (N)
1	20–30	98	51
2	30–40	65	77
3	40–50	55	34
4	50–60	93	21
5	60–70	32	9

**Table 2:** The mean muscle tension values for the male group across different age groups and the selected clinical conditions.

Age Group	No Changes (kPa)	Exudate (kPa)	Arthrosis (kPa)	Disc Displacement (kPa)
20–30	34 ± 5	41 ± 3	25 ± 6	32 ± 3
30–40	27 ± 3	35 ± 4	21 ± 3	20 ± 4
40–50	39 ± 4	40 ± 5	34 ± 4	35 ± 3
50–60	21 ± 2	28 ± 5	37 ± 4	20 ± 4
60–70	18 ± 3	26 ± 4	28 ± 5	20 ± 3

No Changes (kPa): The muscle tension values for males with no visible changes on imaging decreased with age, starting at 34 kPa in the 20–30 age group and gradually declining to 18 kPa in the 60–70 age group. This trend suggests a reduction in baseline muscle tension with aging, possibly due to a natural decline in muscle mass or strength over time. Exudate (kPa): Muscle tension values in the presence of exudate show a similar decreasing trend with age, starting at 41 kPa in the 20–30 age group and declining to 26 kPa in the 60–70 age group. The consistently higher tension values in this condition compared to “No Changes” across all age groups indicate that inflammation, as evidenced by exudate, significantly increases muscle tension. Arthrosis (kPa): The data show varying muscle tension values with age in patients with arthrosis. Muscle tension started lower in the younger age groups (25 kPa in the 20–30 age group), increased in the 40–50 and 50–60 age groups (peaking at 37 kPa), and then declined to 28 kPa in the 60–70 age group. This suggests that arthrosis has a more pronounced impact on muscle tension during middle age. Disc Displacement (kPa): Muscle tension in patients with disc displacement remained relatively stable across age groups, with values ranging from 20 kPa to 35 kPa. There was a slight peak in the 40–50 age group at 35 kPa. The relatively consistent muscle tension values suggest that disc displacement has a steady influence on muscle tension, regardless of age.

**Table 3:** The mean muscle tension values for the female group across different age groups and the selected clinical conditions.

Age Group	No Changes (kPa)	Exudate (kPa)	Arthrosis (kPa)	Disc Displacement (kPa)
20–30	23 ± 4	36 ± 4	17 ± 5	17 ± 3
30–40	27 ± 4	33 ± 4	21 ± 4	20 ± 4
40–50	32 ± 3	37 ± 3	31 ± 3	30 ± 2
50–60	24 ± 4	25 ± 5	27 ± 4	18 ± 4
60–70	14 ± 2	21 ± 3	24 ± 2	17 ± 3

In the “No Changes” category, female muscle tension values started at 23 kPa in the 20–30 age group and decreased to 14 kPa in the 60–70 age group. Compared to males, whose values began higher at 34 kPa and declined to 18 kPa, females consistently exhibited lower muscle tension across all age groups. This may suggest a lower baseline muscle tension in females, potentially due to differences in muscle mass and composition. For the “Exudate” category, females exhibited a decrease in muscle tension from 36 kPa in the 20–30 age group to 21 kPa in the 60–70 age group. Although both genders show elevated muscle tension when exudate was present, females consistently had lower tension values than males (who started at 41 kPa and decreased to 26 kPa). This suggests that while inflammation increases muscle tension in both genders, the magnitude of this increase is generally lower in females.

## Data Availability

The dataset will be provided by the authors on request at r.obuchowicz@gmail.com or gechrzas@cyf-kr.edu.pl.

## Supplementary Materials

The following supporting information can be downloaded at: https://www.mdpi.com/article/10.3390/jcm13175259/s1, Figures S1–S7: The prevalence of unilateral and bilateral occurrence of the disease across age groups.
