# Supplementary material for: Population Analysis of Masseter Muscle Tension Using Shear Wave Ultrasonography across Different Disease States
Source: J Clin Med. 2024 Sep 5;13(17):5259. doi: 10.3390/jcm13175259 (PMC11396082; doi:10.3390/jcm13175259)
Supplement: Supplementary file 1 [file jcm-13-05259-s001.zip › jcm-3181508-supplementary.pdf]

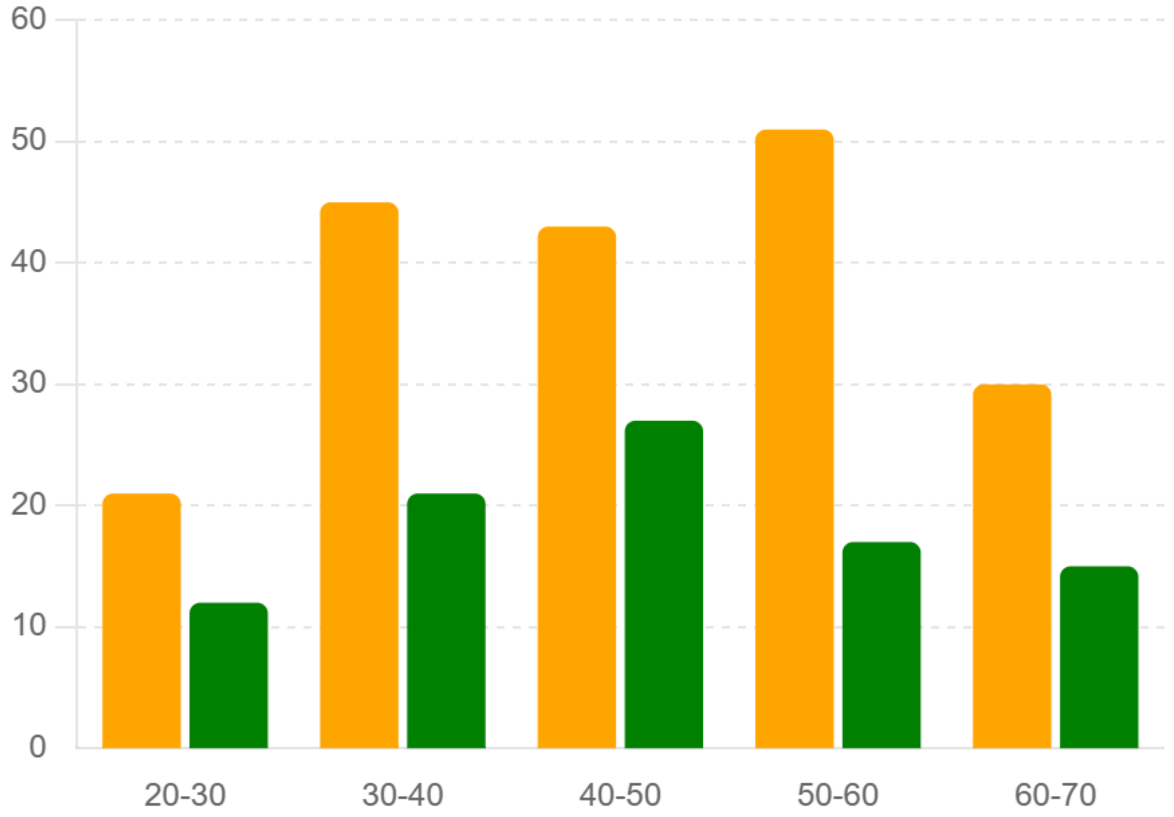

Figure S1. presents distribution go patient number with effusion diagnosed in in the different age groups (five from 20 yo. to 70 yo.) . Unilateral and bilateral effusion in female group is compared side by side marked by orange and green respectively.

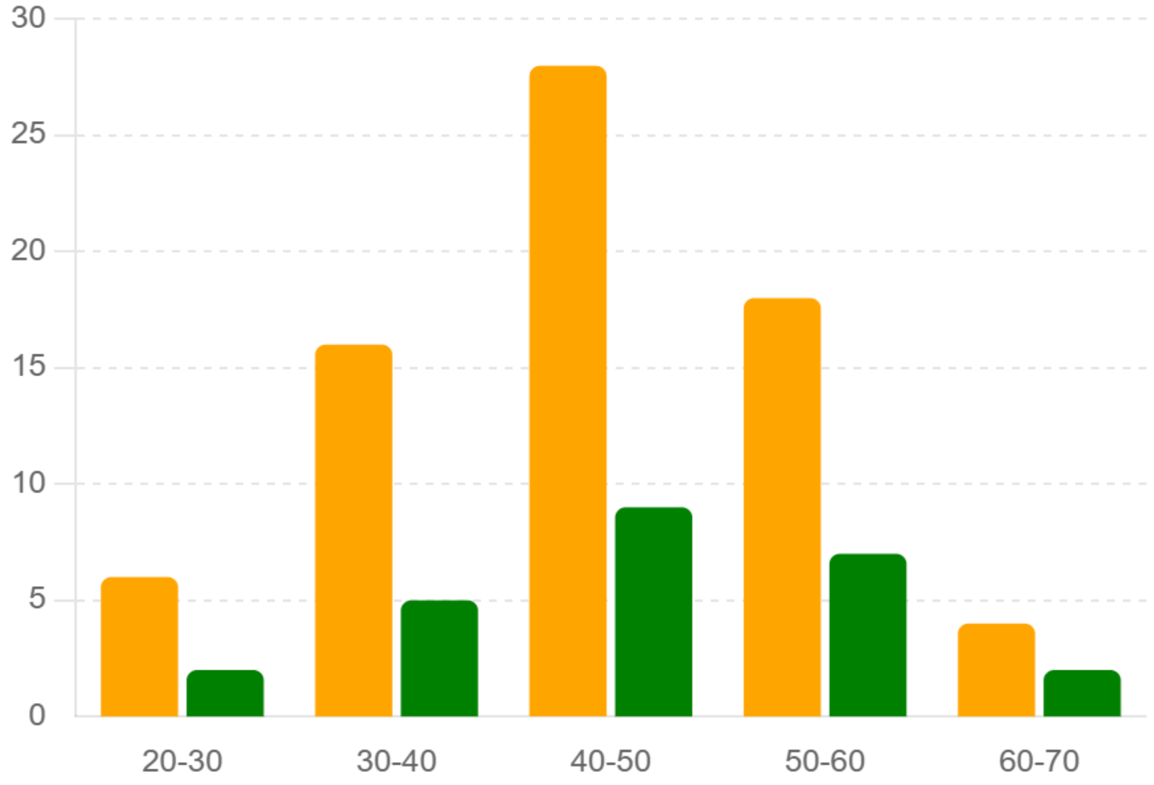

Figure S2. presents distribution go patient number with effusion diagnosed in in the different age groups (five groups from 20 yo. to 70 yo.) . Unilateral and bilateral effusion in male group is compared side by side marked by orange and green respectively.

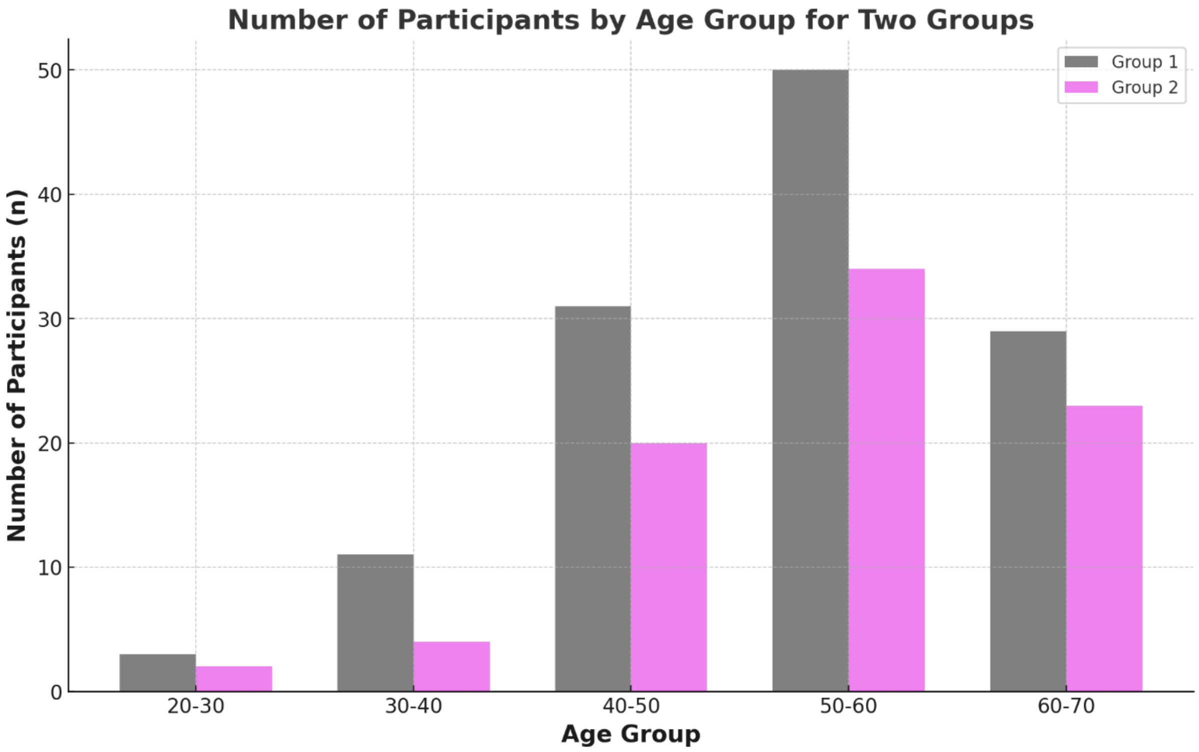

Figure S3. presents distribution go patient number with arthrosis diagnosed in in the different age groups (five groups from 20 yo. to 70 yo.) . Unilateral and bilateral arthrosis in the female group is compared side by side marked by light grey and light violet respectively.

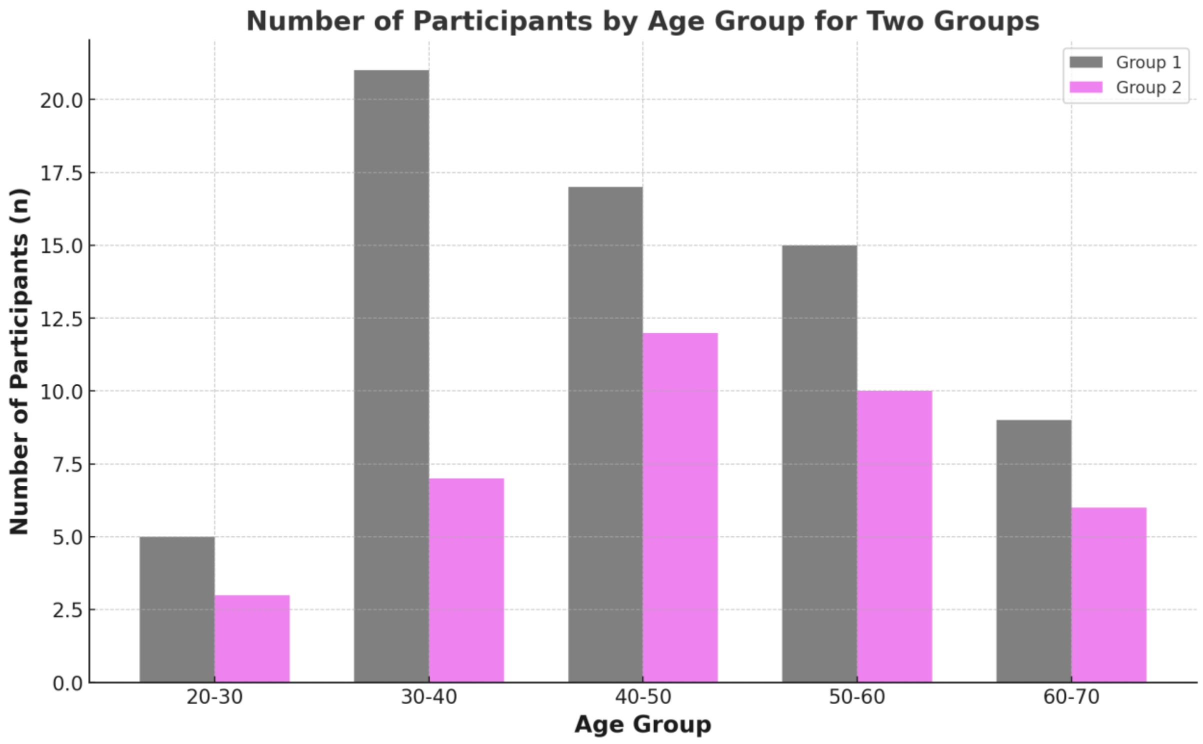

Figure S4. presents distribution go patient number with arthrosis diagnosed in the different age groups (five groups from 20 yo. to 70 yo.) . Unilateral and bilateral arthrosis in the male groups is compared side by side marked by light grey and light violet respectively.

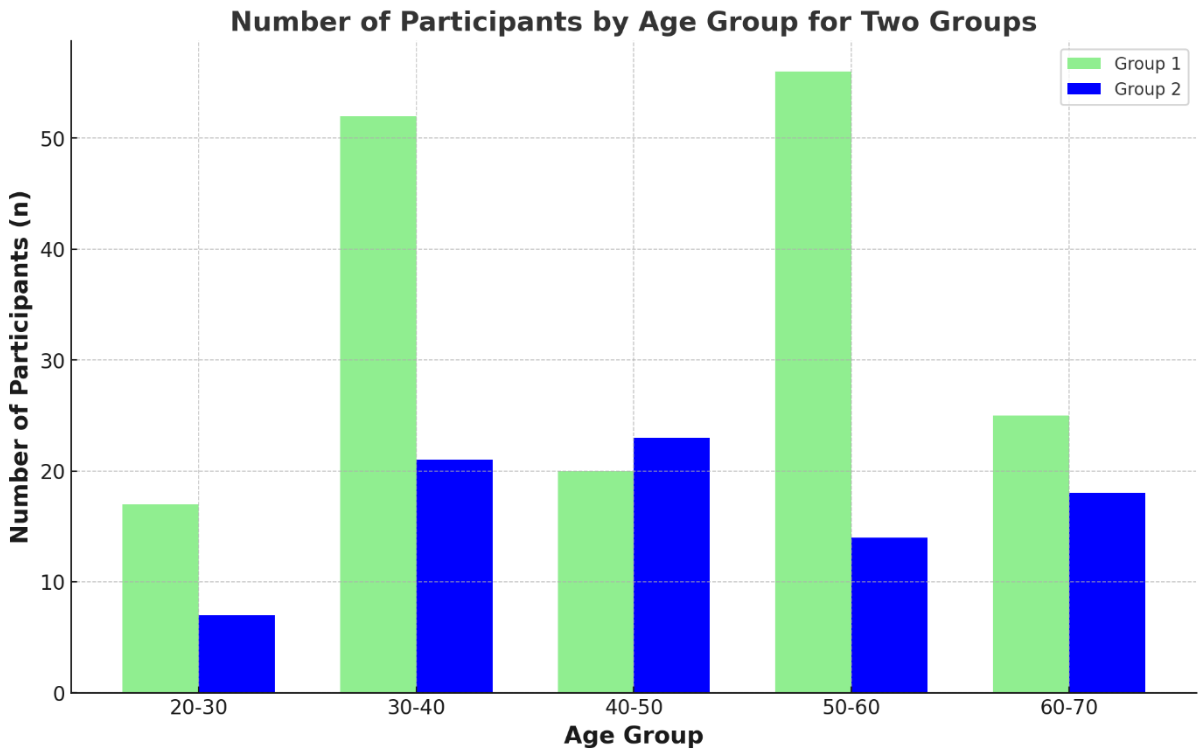

Figure S5. presents distribution go patient number with disk malposition diagnosed in the different age groups (five groups from 20 yo. to 70 yo.) . Unilateral and bilateral disk malposition in the female groups is compared side by side marked by green and blue respectively.

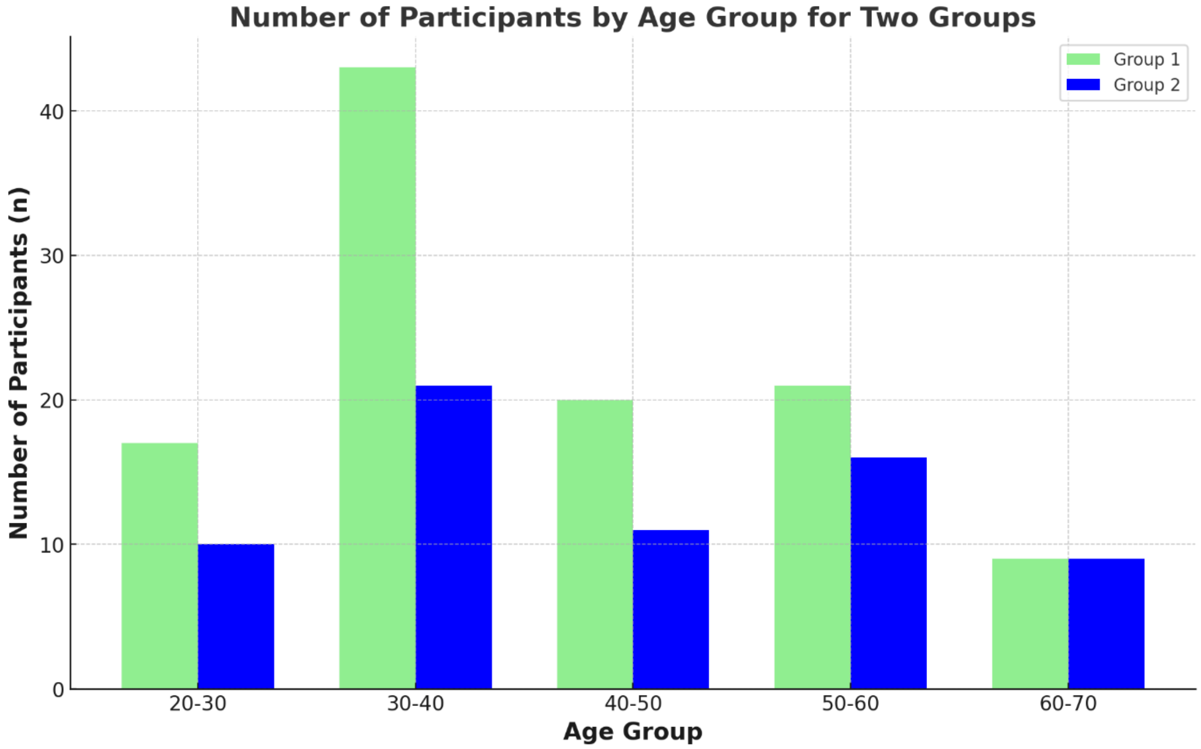

Figure S6. presents distribution go patient number with disk malposition diagnosed in the different age groups (five groups from 20 yo. to 70 yo.) . Unilateral and bilateral disk malposition in the female groups is compared side by side marked by green and blue respectively.

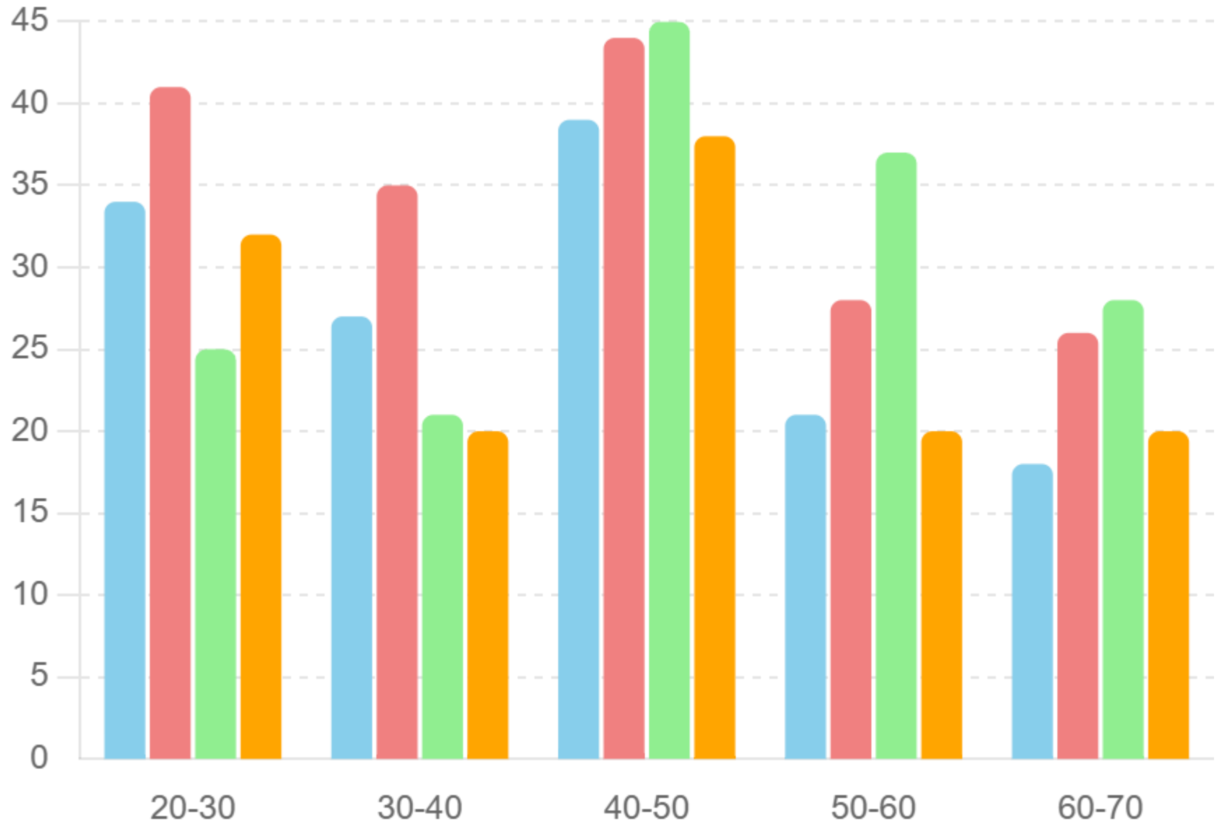

Figure S7. presents dependencies between different muscle tension in the group of female patients with no changes (blue bar), presence of exudate (red bar) , arthrosis (blue bar) and disc displacement (orange bar).
